# Elaboration of an algae‐to‐energy system and recovery of water and nutrients from municipal sewage

**DOI:** 10.1002/elsc.202000007

**Published:** 2020-06-16

**Authors:** Richard K. Laubscher, A. Keith Cowan

**Affiliations:** ^1^ Institute for Environmental Biotechnology (EBRU) Rhodes University Makhanda South Africa

**Keywords:** algae, bio‐energy, integrated algal pond system, mixed liquor suspended solids, sewage

## Abstract

Increasing pressure is being exerted on the peri‐urban space that has elevated the demand for electricity, affects the global water resource, and impacts the potential to produce food, fiber, and commodity products. Algae‐based technologies and in particular algae‐based sewage treatment provides an opportunity for recovery of water for recycle and re‐use, sequestration of greenhouse gases, and generation of biomass. Successful coupling of municipal sewage treatment to an algae‐to‐energy facility depends largely on location, solar irradiance, and temperature to achieve meaningful value recovery. In this paper, an algae‐to‐energy sewage treatment system for implementation in southern Africa is elaborated. Using results from the continued operation of an integrated algal pond system (IAPS), it is shown that this 500‐person equivalent system generates 75 kL per day water for recycle and re‐use and, ∼9 kg per day biomass that can be converted to methane with a net energy yield of ∼150 MJ per day, and ∼0.5 kL per day of high nitrogen‐containing liquid effluent (>1 g/L) with potential for use as organic fertilizer. It is this opportunity that IAPS‐based algae‐to‐energy sewage treatment provides for meaningful energy and co‐product recovery within the peri‐urban space and, which can alleviate pressure on an already strained water–energy–food nexus.

**Abbreviations**
AFP: advanced facultative pond
AP: anaerobic process
HRAOP: high rate algal oxidation pond
HRT: hydraulic retention time
IAPS: integrated algal pond system
IPD: in‐pond digester
MaB‐flocs: microalgal–bacterial floc
MLSS: mixed liquor suspended solids

## 1 INTRODUCTION

By 2050, the human population is forecast to exceed 9.6 billion people. We will require 70% more food [1], 50% more fuel [2], and 50% more potable water [3]. We also need to reduce CO_2_ emissions by over 80% [4]. These targets must be achieved to ensure economic, social, political, climate, food, fuel, and water security. One approach to address the challenge is to recycle water and CO_2_ to fuel and/or chemical products via microalgae using solar irradiance and photosynthesis. Photosynthetic organisms incorporate atmospheric CO_2_ into organic molecules with a net gain in carbon. Indeed, it is well established that 45–50% of microalgal dry matter comprises carbon, and that 1.65–1.83 g CO_2_ is used in the production of 1 g dry microalgal biomass [5, 6]. Therefore, photosynthesis mitigates climate change by counteracting increased levels of atmospheric CO_2_ and the resultant biomass can potentially also provide food and feed, fiber, and even be used in technical processes to generate valuable products including biofuel and clean water.

Microalgae and cyanobacteria are among the most productive photosynthetic organisms on Earth [7]. Microalgae have been reported to obtain higher effective photosynthetic efficiency than energy crops, biomass can be doubled in just a few hours (i.e., as short as 3.5 h) and these microorganisms are believed to synthesize up to 20 times more oil per hectare than terrestrial plants. Furthermore, microalgae can be cultivated on marginal land without affecting food production [8, 9], are able to use industrial flue gas as a carbon source [10, 11], and abstract nutrients from municipal sewage or industrial wastewater [12, 13, 14]. This notwithstanding, researchers have used these microorganisms since the 1950s in an effort to explore and develop systems to mitigate CO_2_ emissions and produce biofuels with particular attention to the development of highly productive bioreactors using genetically manipulated cyanobacteria and algae to produce food and feed, cosmetics, healthcare products or biofuels [6, 11, 15, 16, 17]. However, in most studies thus far, large‐scale production of microalgae biomass appears unsustainable due to the high cost of fertilized media and, the energy required for harvest, dewatering, and processing. Separation of the biomass from the culture medium has proved prohibitively expensive and remains the major limitation in the scale‐up of micro algae bioprocess systems.

Algae‐based sewage treatment was pioneered to bolster more conventional treatment processes such as waste stabilization ponds [18] and today, it is perhaps in the peri‐urban space between sanitation and irrigation where this technology is most needed. Indeed, development and implementation of algae‐based sewage treatment might be crucial in the fight against poor sanitation, waterborne diseases, infections, and contamination of the already limited water resource. In addition to efficient nutrient removal and disinfection, algae‐based bioprocess systems offer the potential for value recovery. In addition to water for recycle and/or re‐use, methane and biomass are typical by‐products. It is these products that are most desired by primary industry (e.g., agriculture and horticulture) in the peri‐urban space and position algae‐based sewage treatment at the water–energy–food nexus.

With ever increasing pressure on the global fresh water resource, derivation of biologically based products particularly food and fuel faces many technical, environmental, and economic challenges. One potential solution is the establishment of algal‐based biorefineries. However, results and economic prognoses from countless studies present a less than favorable outlook and, all efforts to implement algae‐to‐energy wastewater treatment in South Africa has so far been unsuccessful [19]. Contributing factors are many and varied but include the absence of an appropriately defined technological framework, standard but apparently non‐adaptable methodologies, inadequate/incomplete (public) interrogation of the technology, and a disparity of purpose among investors, governance, science‐engineering‐technology service providers, regulatory authorities, and the end user. In this perspective, we elaborate the linkage between algae‐based sewage treatment and energy production to emphasize the net energy that can be gained using an already substantiated integrated algal pond system (IAPS) and the value of its co‐products that include water for recycle and re‐use and an organic nitrogen‐rich liquid fertilizer. Based on real time operation of a 500‐person equivalent (PE) IAPS, energy flows are determined and used to illustrate the potential benefits of this algae‐to‐energy sewage treatment process.

**PRACTICAL APPLICATION**

Increasing pressure is being exerted on peri‐urban spaces that affects global water resources and impacts the capacity to produce food, fiber, and commodity products. Coupling of municipal sewage treatment to IAPS‐based bioprocesses offers potential value recovery. Here, an algae‐to‐energy system is elaborated to show the potential energy and co‐products recoverable in the peri‐urban space. These include: 75 kL per day water for recycle and re‐use,∼9 kg per day biomass for conversion to methane with net energy yield ∼150 MJ per day, and ∼0.5 kL per day high nitrogen‐containing liquid fertilizer.

## 2 ALGAE‐BASED WASTEWATER TREATMENT

Microalgae have been associated with the treatment of various wastewaters, including sewage, using processes ranging from traditional oxidation ponds (or waste stabilization ponds) to more advanced high rate algal oxidation ponds (HRAOPs). These ponds have in common a functional microbial consortium comprising of micro flora and fauna that together treat wastewaters [20]. More specifically, HRAOPs are shallow, paddlewheel‐driven raceways that facilitate high photosynthetic activity and algal growth, elevated pH, and supply dissolved oxygen (up to three times saturation) for heterotrophic bacteria that break down remaining dissolved organic matter. The effectiveness of HRAOPs for wastewater treatment is quite well documented and has been demonstrated up to full commercial scale [21, 22, 23, 24]. Implementation of HRAOPs is also considered by many to be a cost‐effective means of upgrading waste stabilization ponds to increase treatment capacity by incorporating these into existing infrastructure [25, 26]. Additionally, HRAOPs have been proposed in the managed cultivation of microalgae for downstream processing in biorefineries from which nutrient‐rich biomass can either be beneficiated for use as fertilizer or, processed as feedstock for bulk commodity products including biofuels [25, 27, 28, 29].

**FIGURE 1:**
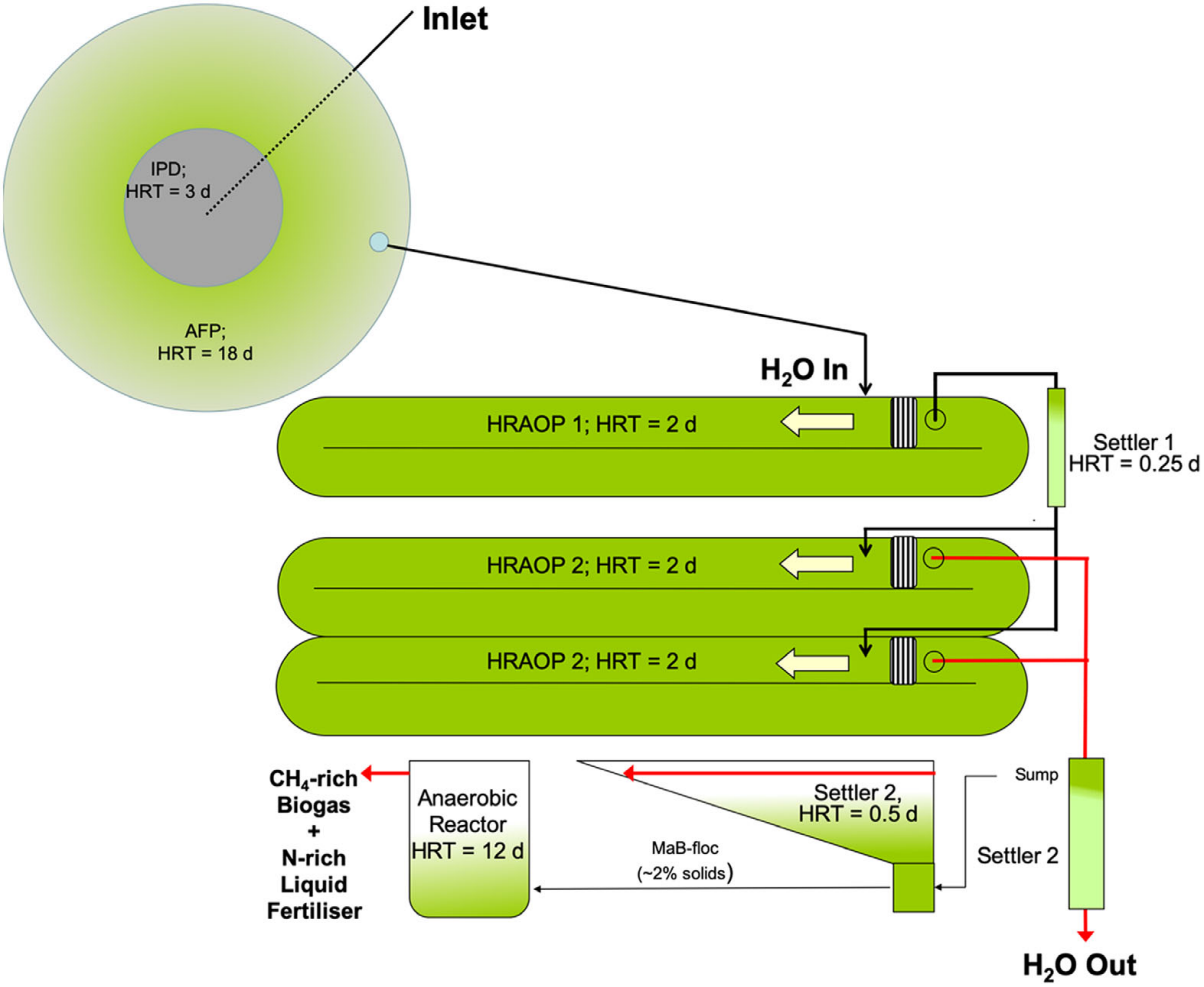
Schematic illustrating the pilot‐scale 500‐person equivalent algae‐to‐energy system for municipal wastewater treatment and recovery of treated water, biomethane, and nitrogen‐rich fertilizer. Located at the Institute for Environmental Biotechnology Rhodes University (EBRU), Makhanda (33°19′ 07″ south, 26° 33′ 25″ east), South Africa, the IAPS described here operates continuously and consists of an advanced facultative pond (AFP) that incorporates an in‐pond digester (IPD) and a series of high rate algal oxidation ponds (HRAOP) connected by settlers. Raw domestic effluent is introduced into the system via the IPD ∼6 m below surface where anaerobic digestion of biosolids takes place. Effluent from the IPD/AFP gravitates to a series of HRAOP in which constant mixing is carried out by a paddlewheel generating a linear velocity of 0.3 m/s. Mixed liquor containing the MaB‐flocs flows via the settler wherein the biomass is passively settled and recovered as a slurry concentrated to 2–3% solids for conversion to biomethane and fertilizer. Treated effluent from the IAPS is discharged to a maturation pond series. HRT, hydraulic retention time. Note: schematic is not to scale

The concept of integrating different ponds (anaerobic, facultative, oxidation, and high rate algal oxidation ponds) to achieve biological wastewater treatment was developed by Oswald and co‐workers in the late 1950s. Today, the process is trademarked as AIWPS^®^ according to a U.S. Environmental Protection Agency report [30] and in New Zealand, is being implemented as the enhanced pond system (EPS) technology [31]. This concept technology has also been innovated and configured for southern African conditions as the integrated algal pond system (IAPS). As an amalgamation of anaerobic and aerobic biological processes the process comprises the following: advanced facultative pond (AFP) that incorporates a fermentation pit or in‐pond anaerobic digester (IPD) followed by a series of HRAOPs and settlers. In the AFP, the innovative design of the digester ensures complete breakdown of bio‐degradable solids, including parasites (e.g., helminthic ova, worms, etc.) which therefore eliminates the need to heat the pit or handle and dispose of sludge. In addition, heavy metals remain in the fermentation pit and are typically precipitated as metal sulfides and/or insoluble salts. The bottom layers of the AFP are anaerobic and/or anoxic and are overlain by oxygen‐containing layers rich in algae and bacteria. These organisms sequester CO_2_, and oxidize residual methane, hydrogen sulfide, and nitrogen, produced during fermentation. Excess effluent from the AFP flows into a series of HRAOP where it is subjected to photosynthetic oxygenation. Under optimal conditions most of the dissolved nutrients are assimilated into a biomass or mixed liquor. At hydraulic retention time of ∼4 days, complete disinfection is accomplished through elevated pH and oxygen, and by exposure to UV radiation from sunlight, even in winter. The biomass produced in the HRAOP is easily and continuously removed by passive settling to yield ∼27 000 kL per annum of high‐quality water for recycle and/or re‐use and the complete process is delineated in Figure 1. A detailed re‐evaluation of this technology confirmed that the treated water complies with the South African general limit values for either irrigation or discharge into a water resource that is not a listed water resource for volumes up to 2 ML of treated wastewater on any given day [32].

## 3 ALGAE‐DERIVED RENEWABLE ENERGY

Mixed liquor suspended solids (MLSS) is the term used to describe the concentration of suspended solids in the aeration tank during the activated sludge process. It comprises microorganisms and non‐biodegradable suspended matter and ensures that sufficient active biomass is available to consume the quantity of organic pollutant supplied. Similarly, HRAOPs of IAPS (and AIWPS and EPS) can be thought of as aeration tanks in which oxygen is introduced, not by mechanical means (e.g., activated sludge) but by algal photosynthesis, to ensure consumption of organic pollutants.

The ability of a system to introduce oxygen into a mixed liquor is the oxygenation capacity (OC) of the system whereas the energy required is the oxygenation efficiency (OE). In general terms, aeration systems are compared on the basis of OE, typically expressed as the amount of oxygen introduced per unit of energy expended (kgO_2_/kWh). Several studies indicate that total OC of HRAOPs taken together with the installed power for paddlewheels gives an OE of 15 kgO_2_/kWh. Since mechanical aerators rarely transfer oxygen from air to water at more than 1 kg/kWh, photosynthetic oxygenation of MLSS appears to be ∼10 times more efficient emphasizing its importance in algae‐based wastewater treatment.

**FIGURE 2:**
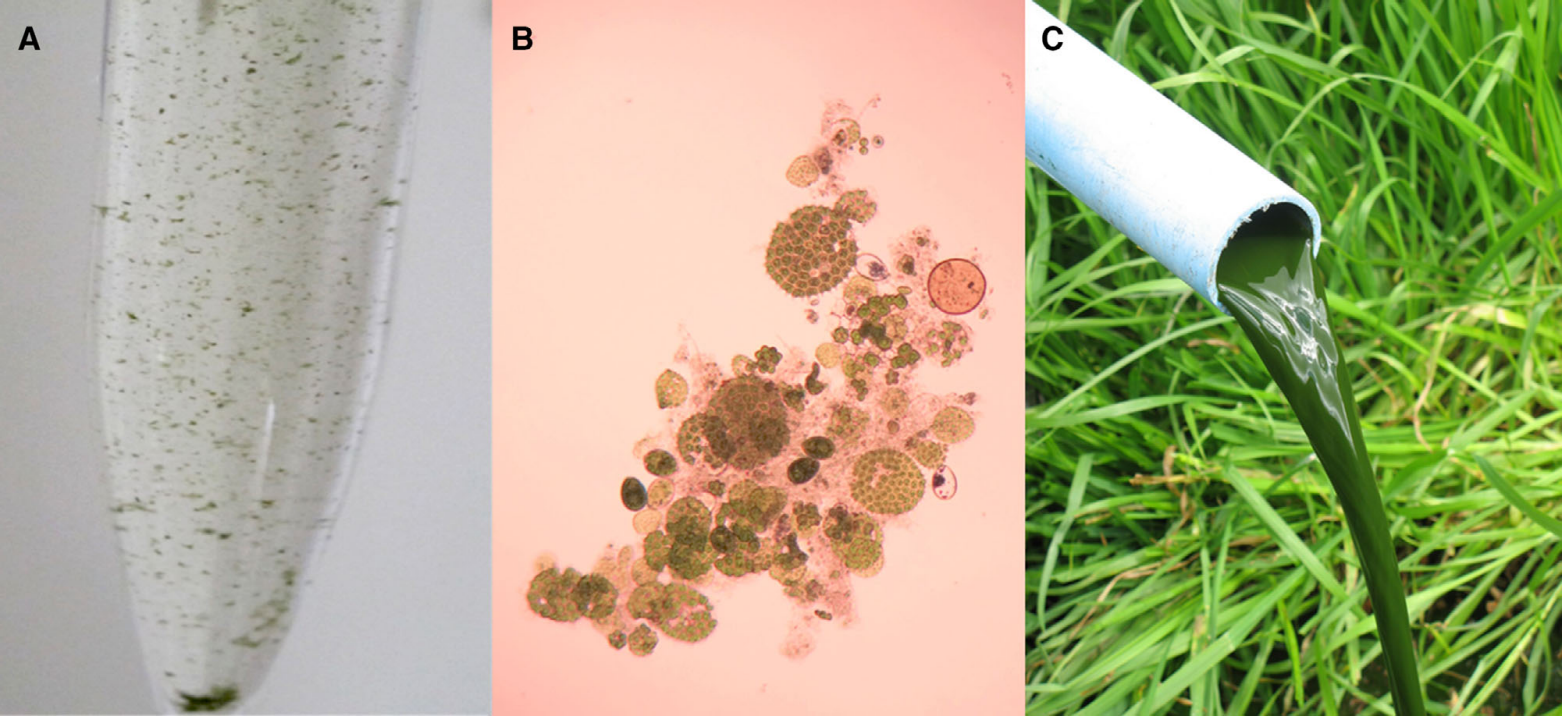
Composition of mixed liquor suspended solids (MLSS) in high rate algal oxidation ponds. Sample of MLSS from the HRAOPs of a 75 000 L per day IAPS treating municipal wastewater, (A); light micrograph (×40 magnification) of a single MaB‐floc contained in the mixed liquor (B); and, settled biomass slurry (∼2% solids) for transfer to anaerobic reactor (C)

Integrated algal pond system HRAOPs therefore act as continuous stirred tank reactors and, flow through these is governed by a hydrostatic head. For reasons outlined above, biomass in the HRAOPs can be regarded as MLSS that in this instance is composed predominantly of microalgae in association with bacteria in a floc constructed of extracellular polymers [33]. Indeed, it is the continuous exchange of O_2_ and CO_2_ between microalgae and bacteria that facilitates microalgae–bacterial floc (MaB‐floc) formation [34, 35, 36]. And, as described below (see Figure 2), it is formation of these MaB‐flocs that accelerates nutrient removal from wastewater, increases productivity, facilitates passive settling, ease of harvest, and consequently, conversion of the biomass into renewable energy.

A recent study indicated a positive correlation between microalgal cell degradation and the amount of biogas produced [37]. Species lacking a carbohydrate‐based cell wall (e.g., *Dunaliella salina*) or those with protein‐based cell walls lacking hemicellulose or cellulose (*Chlamydomonas reinhardtii*, *Arthrospira platensis*, *Euglena gracilis*) have elevated methane generating potential. In contrast, *Chlorella kessleri*, *Nannochloropsis salina*, and *Scenedesmus obliquus* have carbohydrate‐ and/or algaenan‐based cell walls containing hemicellulose and hydrophobic ether‐crosslinked saturated aliphatic chains, respectively, which limits biochemical methane yield. Thus, various pretreatment steps might be required to enhance biomass hydrolysis for conversion to methane. It is therefore perhaps to be expected that MaB‐Flocs from municipal wastewater after enzymatic pretreatment produced biogas with higher quality methane (68–72%) and, with maximum biogas yield of 369.44 ± 6.36 mL/g volatile solids (VS) under mesophilic conditions [38]. By comparison, none of the pretreatment methods tested improved methane yield from MaB‐floc feedstock produced in aquaculture wastewater that led to the conclusion that MaB‐floc biomass should be granted only a supporting role within a biorefinery [39]. Thus, methane production from microalgae biomass is currently considered neither profitable nor sustainable unless part of a process such as wastewater treatment [40]. Harvesting aside, major limitations to the use of microalgae appear to include the need for pretreatment, seasonal variation in quantity and quality of biomass, and the need to improve biochemical methane yield [41].

## 4 FACTORS AFFECTING ENERGY FLOW IN AN ALGAE‐TO‐ENERGY SYSTEM

Focused mass culture of microalgae has traditionally been high‐value product‐ and species‐driven and, carried out without due consideration of the many variables that are evident in an algae‐to‐energy process. A move toward production of MaB‐floc biomass as part of a municipal wastewater treatment process in peri‐urban areas for recovery of treated water and energy, necessitates detailed decision‐making prior to design and implementation.

The focal point for any algae‐based wastewater treatment process is the geography in which production and processing of MaB‐floc biomass will take place. Typically carried out to render treated water safe for disposal, without risk to public health and without pollution of receiving watercourses, wastewater treatment plants are located at or near the lowest point in the peri‐urban zone. For many municipalities, it is becoming increasingly important that nutrients, energy, and other valuable resources in addition to water, be recovered from the wastewater treatment process. Producing biomass as an outcome of wastewater treatment is one example and for this, factors that impact operational efficiency will exert a profound impact on yield. Of primary importance is solar irradiance as this parameter has a major influence on the water treatment efficacy of algae‐based systems. In addition, climatic factors such as temperature, biotic factors, wastewater volume and composition, whether Green‐ or Brownfield installation, available waste streams and, end user (e.g., agriculture) needs, all impact the decision‐making process. Most critical, however, is solar irradiance (and ambient temperature).

### 4.1 Solar irradiance

While installation of wastewater treatment infrastructure (i.e., Greenfield) or increasing the capacity of existing structures (i.e., Brownfield) provides opportunity and access to land, it is solar irradiance that determines whether an algae‐to‐energy bioprocess can indeed be successfully implemented. Productivity in HRAOPs together with heterotrophic bacterial action, EPS production, and MaB‐floc formation is the outcome of photosynthetic carbon assimilation and also driven by solar irradiance. Of the total amount of incident solar irradiance only light in the 400–700 nm region of the electromagnetic spectrum is used for photosynthesis. Termed photosynthetically active radiation (PAR), the actual quantity of total irradiance used in photosynthesis has been estimated at ∼48%. Maximum conversion efficiency of solar irradiance into biomass remains a controversial topic and recent estimates vary widely. Even so, for land plants, photosynthetic efficiency is generally accepted to be 4.6% for C_3_ plants and 6.0% for C_4_ plants [42]. For microalgae in wastewater treatment systems, predation and low nutrient concentration also contribute to reduced overall efficiency of light conversion into chemical energy. Furthermore, >60% of light absorbed by MaB‐flocs in HRAOPs can be dissipated as heat that also reduces photosynthetic efficiency [43]. Thus, most microalgae in open HRAOPs and under natural irradiance, are unlikely to sustain a photosynthetic efficiency more than 4–5% [44].

Although many researchers assume a mean global daily irradiance, usually for a defined geography (e.g., central Europe), this can be problematic. For example, six zones of irradiance have been delineated for South Africa [45]. These range from 123.8 to 140 W/m^2^ along the southeast coast to 263–278.4 W/m^2^ in the northwest arid region. Although the long‐term mean annual global irradiance for South Africa has been estimated as 234.5 W/m^2^, it is perhaps not advisable to use average irradiance across isophotes to derive mean global daily irradiance. Rather, solar irradiance for a specific location should be derived based on measurement of surface annual averaged global irradiance for the location in question. This will ensure that maximum possible photosynthetic efficiency and thus nutrient abstraction, MLSS production and wastewater treatment will be attainable for a bespoke algae‐to‐energy system.

Very often there is confusion surrounding the terms light intensity, irradiance, and photon flux density and these are sometimes used interchangeably that is incorrect. To clarify, light intensity or illuminance (with SI unit lux) is the total luminous flux incident on a surface per unit area and entirely dependent on external circumstances. For example, light distributed over 16 m^2^ may have intensity 53 lux whereas the same light in a 1 m^2^ area gives 850 lux. By comparison, irradiance of a light source is defined as the power of electromagnetic radiation incident per unit area of surface and has the SI unit W/m^2^. For biological processes, the quantum flux of light with a distinct wavelength, typically visible light in the 400–700 nm range, has a higher relevance than irradiance. Quantum flux (sometimes called photon flux) is defined as the number of photons of light per second per unit area of surface and has the unit, µmol m^−2^ s^−1^.

### 4.2 Resource availability

In addition to availability of large volumes of nutrient‐containing wastewater and sufficient land, the availability of CO_2_‐rich flue gas and waste heat can make a considerable contribution to the overall cost‐benefit of implementing an algae‐to‐energy wastewater treatment system.

Since CO_2_ is limiting in photosynthesis, enrichment of HRAOPs with flue gas‐derived CO_2_ may increase MLSS productivity and contribute to more efficient nutrient removal, OE, MaB‐floc yield, water treatment, and a better‐quality final effluent either for recovery, discharge to the environment, or irrigation. Furthermore, seasonal variations in climate impact biological wastewater treatment processes and are factored into the design and operation prior to implementation. Production of MLSS and hence MaB‐floc biomass in HRAOPs depends on the interplay between average incident irradiance, gas exchange (CO_2_ supply, O_2_ removal), nutrient concentration, and temperature. This interplay can be severely affected by exposure to extreme temperature fluctuation and particularly to sustained low temperature. So, waste heat can be used to mitigate the effects of low temperature by maintaining conditions that favor MaB‐floc biomass production and its conversion to methane.

### 4.3 Carbon:nitrogen ration

Another critical consideration in determining the suitability of a feedstock for conversion to CH_4_ is its carbon:nitrogen (C:N) ratio. Feedstock with high protein content can result in release of substantial quantities of NH_3_ and, although acclimation is possible, reduced CH_4_ output is a likely consequence. Although there is little or no published data available, CH_4_ yield from MaB‐flocs or the dominant species within these flocs is dependent on both species’ composition and cultivation conditions. Typically, the C:N ratio for microalgae is ∼6 [40] and for HRAOP MaB‐floc biomass, values close to 6 have been documented [38, 39]. In the example presented here, C:N values ≥12 are usual. This higher C:N ratio of MaB‐floc biomass is attributed to maintenance of a low nitrogen concentration in HRAOPs by removal of biomass during operation of the IAPS wastewater treatment process [24]. Even so, addition of non‐proteinaceous organic materials to the anaerobic reactor can help avoid NH_3_ inhibition. Yen and Brune [46] demonstrated that CH_4_ production could be enhanced twofold when an equal amount of waste paper was co‐digested with algal sludge compared to pure algae slurry at identical loading rates. These authors established that a C:N ratio of 20:1 to 25:1 was optimal, which is possible if other low nitrogen containing organic resources such as food and food processing waste are available as co‐feedstock.

## 5 ANAEROBIC DIGESTION AS THE METHOD OF CHOICE

Notwithstanding the above, microalgae and by inference, HRAOP‐derived MaB‐flocs, fulfil all of the nutritional requirements, including supply of essential trace elements needed by an anaerobic consortium [47]. Whereas many studies indicate recalcitrance of microalgae to hydrolysis and conversion to methane, results using tailored anaerobic microbiomes suggest that pretreatment may not always be necessary [48]. Similarly, feedstock comprising substantial quantities of easily accessible carbon such as extracellular polymeric substance (EPS)‐rich MaB‐floc‐containing biomass may surpass pure microalgae biomass as feedstock. Formation and aggregation of microalgae and bacteria into MaB‐flocs as an outcome of EPS production by these microorganisms in HRAOPs has recently been demonstrated [49] and appears to arise for the purposes of chemical and developmental interaction, protection, communication, aggregation, and adhesion [33]. Typical MaB‐flocs produced in HRAOPs of an IAPS treating municipal sewage are shown in Figure 2A and B.

Anaerobic conversion of biomass to biomethane is a four‐stage process and is carried out by a consortium of fermentative and, acetogenic and methanogenic bacteria. In brief, the first stage involves chemoheterotrophic bacteria that hydrolyze complex biopolymers to oligomeric and monomeric sugars, peptides, and amino acids. In stage 2, short chain volatile organic acids are formed in a process called acidogenesis and in stage 3, obligate acetogens convert these to H_2_, CO_2_ and acetate in a process referred to acetogenesis. In stage 4, the conversion of acetate to CO_2_ and CH_4_ occurs due to both acetoclastic and CO_2_‐reducing methanogenic bacteria [47, 50, 51].

Using published values for percentage energy flow from complex materials through the various stages of the anaerobic process and the molecular formula of the feedstock, its potential for biochemical conversion to CH_4_, CO_2_ and NH_3_ can be summarized as follows:

$$\begin{array}{ccc} C_{a}H_{b}O_{c}N_{d} & + & \frac{4a-b-2c+3d}{4}H_{2}O\to \frac{4a+b-2c-3d}{8}CH_{4}\\ & + & \frac{4a-b+2c+3d}{8}CO_{2}+\mathrm{dN}H_{3}\; \end{array}$$

For microalgae biomass with formula CO_0.48_H_1.83_N_0.11_P_0.01_ [52], 51% can in theory be converted to CH_4_. However, chemical composition of microalgae‐containing biomass is influenced by species, growth conditions, nutritional status, and oxidation state. Consequently, concentration of CH_4_ in the biogas produced can vary from 46 to 76% (v/v) while the ratio of CH_4_ to CO_2_ is a result of feedstock oxidation state. Even so, theoretical methane yield (B_0_) can be calculated from a known VS content using Equation 1:

(1) $$B_{0}=\frac{4a+b-2c-3d}{12a+b+16c+14d}V_{m}\;$$

where a, b, c, and d are the molar ratios of C, H, O, and N, respectively; and V_m_, is the normal molar volume of methane at standard temperature and pressure and equivalent to 22.4 L CH_4_ per gram VS.

Solubility of CO_2_ in aqueous media and its partitioning into carbonate might be expected to impact composition of recovered biogas. For example, calcium carbonate is one of the minerals that naturally precipitates as a by‐product of microbial metabolic activity [53], and as a consequence, actual amount of biogas produced may be less than the theoretical yield. Also, a portion of feedstock is used to fuel growth of the anaerobic bacterial consortium and this is estimated at between 5 and 10% while ∼10% of the original feedstock, remains undigested [51].

To illustrate the potential of the anaerobic process for use in an algae‐to‐energy wastewater treatment system, energy flows were approximated using real time data from mixed liquor (i.e., MaB‐floc biomass) produced in HRAOPs of an IAPS treating municipal sewage.

## 6 ENERGY FLOW IN AN ALGAE‐TO‐ENERGY WASTEWATER TREATMENT SYSTEM

For production of HRAOP‐derived MLSS and conversion to CH_4_, the model described herein has used real‐time operation and production data from a technical‐scale IAPS wastewater treatment system and the associated mass and energy flows are delineated in Table 1.

**TABLE 1:** Mass and energy flows in the 500 PE IAPS algae‐to‐energy system

HRAOP dimensions and productivity			Comments
Surface area	1000	m^2^	Combined surface area of two identical HRAOP
Volume	300	m^3^	Volume maintained by continuous flow i.e. inflow = outflow
Depth^a^	0.3	M	Depth maintained by raised standpipe
HRT	4	D	Period effluent is detained in HRAOP
MLSS	0.154	g/L	Average MLSS (August to November, Southern Hemisphere)
Productivity^b^	0.036	kg/ha per day	Average productivity (August to November, Southern Hemisphere)
**Per diem mass and energy flows from HRAOP to AP**			
Biomass discharged	11535	g per day	Average dry biomass from HRAOP to settler
Biomass settled	9228	g per day	Average dry biomass settled and pumped to anaerobic reactor
Residual MLSS (or suspended solids)	0.031	g/L	Average suspended solids in treated wastewater
Paddlewheels	64.8	MJ per day	Electrical power required to circulate effluent in HRAOP
Pumping	1.7	MJ per day	Electrical power required to transfer biomass to reactor
**Per diem flow from HRAOP to AP**			
Volume of pond decant	75000	L per day	Volume of treated wastewater from HRAOP to settler
Volume of slurry	461	L per day	Volume of collected biomass (∼2% slurry) for transfer to anaerobic reactor
Volume of supernatant	74539	L per day	Volume discharged to tertiary treatment
**Anaerobic process**			
Lower heating value of methane^c^	35.92	MJ m^‐3^ CH_4_	
Methane potential of HRAOP biomass	0.662	m^3^ CH_4_ kg^–1^	Methane potential of MaB‐floc biomass with chemical formula C_12_H_23_O_4_N
Methane produced from biomass daily	6.109	m^3^ CH_4_ per day	Methane produced from settled biomass
Energy output	219.4	MJ per day	Per diem energy output calculated using LHV of methane
Net energy	153.0	MJ per day	Per diem energy recovered after deduction of electrical power input

The IAPS used in this study is located at the Belmont Valley Municipal WwTW, Makhanda, South Africa, in an isophote with 201.3–216.7 W/m^2^ annual averaged solar irradiance, supplied 75 000 L per day municipal wastewater and, configured and operated as described elsewhere [24, 49]. See text for description of component parts and operation and, for derivation of energy flows.

a Sleeve inserted into the pond floor drain to raise drainage point by 0.3 m

b Productivity calculated from MLSS determinations using formula *P* = 10^d/t^ n × MLSS

where P, productivity (kg/ha per day); d, pond depth (m); t, hydraulic retention time (day); MLSS, mixed liquor suspended solids (mg/L); n, algae ratio in the MLSS (0.9–1.0) as described by Al‐Shayji *et al* [55].

c Lower heating value (also known as net calorific value) is the amount of heat released by combusting a specified quantity (initially at 25°C) and returning the temperature of the combustion products to 150°C, which assumes the latent heat of vaporization of water in the reaction products is not recovered.

In this system, the HRAOP component of the IAPS comprises two identical raceway‐type ponds, each of 500 m^2^, 0.3 m operating depth, and volume 150 kL. The content of each pond is mixed continuously by an in‐pond paddlewheel, driven by a 0.375 kWh motor, with 4 day hydraulic retention time (HRT), and the volume of treated water discharged is 37.5 kL per day. All HRAOP water exits the process via a settler tank with HRT of 0.5 day. Settled biomass is collected in the settler tank sump from where it can be transferred to the anaerobic reactor for processing to CH_4_. Treated water is either subject to peroxonation for irrigation purposes or discharged after chlorination. If required, a portion of the settled biomass can be beneficiated to high‐value and/or other commodity products. In this example, all of the recovered biomass is used as a substrate for conversion to CH_4_.

Combined late winter and spring data revealed an average pond MLSS concentration of 153 mg/L with daily productivity of 36 mg/L per day (∼10.8 g/m^2^ per day) and an amount of biomass entering the settling tank equivalent to 11.5 kg per day. During this period, typically associated with low photosynthetic activity and productivity in aquatic systems, total daylength was ∼10 h in August and increased to ∼12 h by November and, mean daily minimum and maximum temperature was 7/18°C in August and increased to 13/22°C in November.

With 80% of the MaB‐flocs settled passively, 9.2 kg accumulated each day in each settler tank. This should yield a total settled MaB‐floc biomass with volume ∼0.46 kL per day and solids content ∼2% (w/v). A 1 kWh submersible pump with a throughput of 1 kL/h is used to transfer the settled biomass to the anaerobic reactor and the energy consumed in the transfer process is dependent on the volume of slurry to be transferred (Figure 2C).

Elemental analysis of MaB‐floc biomass indicated a molecular mass of C_12_H_23_O_4_N with a C:N ratio of 12:1 and, much higher than reported for microalgae biomass. As outlined above, a higher C:N ratio reduces the amount of ammonia produced during the anaerobic process and the risk of ammonia toxicity. The stoichiometry of the anaerobic process for the MaB‐floc biomass can therefore be summarized as follows:

$$2C_{12}H_{23}O_{4}N+10H_{2}O\to 15CH_{4}+9CO_{2}+2NH_{3}$$

As described by Buswell and Neave [54] and, as recommended by others [47, 51], Equation 1 can be used to determine the theoretical volume of CH_4_ that can be produced from volatile solids.

From Equation 1, it becomes apparent that a theoretical yield of 1 g of VS will yield 0.66 L of CH_4_. However, theoretical yields are not easily achieved for the following reasons: some organic material is recalcitrant and avoids hydrolysis, a portion of the inflow into the anaerobic reactor passes through unprocessed, and the bacterial consortium utilizes a portion of the volatile solids for cellular growth. If 10% of volatile solids is allocated to each factor, then an efficiency of conversion of 73% is realized. Consequently, the theoretical yield discounted at 73% will yield 0.48 L of CH_4_ per gram VS. Thus, the settled MaB‐flocs introduced into the anaerobic reactor would yield around 6.1 m^3^ of CH_4_ per day with the anaerobic digester at steady state. Using the lower heating value (LHV) of 50.1 MJ/kg and density of 0.72 kg/m^3^ for CH_4_, it is estimated that MaB‐floc biomass produced in HRAOPs of an IAPS treating municipal sewage yields an energy output equivalent to 219 MJ per day. Since the treatment of domestic wastewater and the production of MaB‐flocs in the HRAOPs does consume energy, specifically 66 MJ per day for operation of paddlewheels, and a further 1.7 MJ per day for the transfer pump, these energy inputs need to be reconciled with the energy yield from CH_4_ produced. A positive net energy output of 153.0 MJ per day is therefore possible from this 500‐PE, algae‐based wastewater treatment system (Table 1).

Ammonia released by anaerobic decomposition of organic matter can be calculated using the following equation [47]:

(2) $$Y_{N-NH_{3}}=\frac{d\times 17\times 1000}{12\times a+b+16\times c+14\times d}$$

Referring to the molar ratios of the elemental composition of HRAOP‐generated MaB‐flocs as indicated above, a theoretical NH_3_ yield in the anaerobic process of 69.4 mg/g VS seems possible. At steady state, the anaerobic reactor receives 2% settled MaB‐floc with dry weight of approximately 9.22 kg every day and, with an efficiency of 73%, the per day NH_3_ yield is ∼471 g. Assuming the volume of effluent leaving the reactor is similar to the volume of settled MaB‐floc supplied (i.e. 0.46 kL per day), corrected for water consumed in the anaerobic process, the concentration of ammonia nitrogen in the digestate would be ∼1.02 g/L. Ammonia nitrogen accumulated in the anaerobic process exists largely in two forms; as the ammonium ion (NH_4_^+^) and as free ammonia (NH_3_). Using percentage NH_3_ in an aqueous solution as a function of temperature and pH as determined by Emerson and co‐workers [56] and, assuming a reactor pH of 7 at 20°C, only 0.39% of the ammonia would be in the un‐ionized form with >99.9% as NH_4_^+^, which is not easily volatilized and less toxic to the consortium and the anaerobic process.

The volume of effluent discharged from the anaerobic reactor is small in comparison to the 75 kL volume of treated wastewater discharged daily from the HRAOPs of this IAPS. One possible end use for this nutrient‐rich fraction is as a liquid fertilizer and/or biofertilizer [57]. Indeed, research has moved away from fossil fuel as the source material for fertilizer toward exploitation of microbes as less environmentally damaging and sustainable approach [58, 59]. Thus, production of organic liquid fertilizers of the NPK type are distinctly possible and deserve to be considered as outcomes of algae‐to‐energy wastewater treatment.

## 7 CONCLUDING REMARKS

An algae‐to‐energy system that leverages off wastewater treatment overcomes the major constraints of cost of land and nutrients needed for mass production. Further benefits may accrue should the facility be located in a region that receives adequate solar irradiance, has ambient temperature close to optimum for microbial growth and activity (both IAPS and anaerobic reactors), and if additional resources such as high carbon containing organic waste, flue‐gas derived CO_2_, and heat are available. Although energy cost associated with separating algae from water has always been a major obstacle in the design and implementation of algae‐to‐energy systems, production of MaB‐flocs by a mixed consortium within HRAOP of IAPS facilitates settling and simplifies the process of biomass recovery. These flocs have many attractive properties, namely their propensity to self‐flocculate and settle rapidly without the addition of flocculants, and their adequacy as feedstock for anaerobic digestion. In the latter case, the process design of the IAPS produces a biomass with a C:N ratio of 12:1, double that of most algae culturing systems, which makes it more amenable to anaerobic digestion due to lower ammonia production. As a consequence, a net energy gain appears to be the outcome of utilizing IAPS as an algae‐to‐energy wastewater treatment system. Use of gravity flow in wastewater treatment together with microbial production of extracellular polymers, formation of MaB‐flocs and harvest by passive settling of biomass, mitigate operational costs in providing feedstock for bio‐conversion to CH_4_ and production of a nitrogen‐rich humic containing liquid organic fertilizer. Realization of this opportunity for meaningful energy and co‐product recovery within the municipal peri‐urban space can offset mounting pressure on the water‐energy‐food nexus.

## CONFLICT OF INTEREST

The authors have declared no conflict of interest.
